# Saunders’s Medical Hand-Atlases

**Published:** 1905-05

**Authors:** 


Saunders's Medical Hand-Atlases. Atlas and Epitome of Operative Ophthalmology. By Dr. O. Haab, of Zurich. Edited, with additions, by George E. de Schweinitz, M. D., Professor of Ophthalmology in the University of Pennsylvania. With 30 colored lithographic plates, 154 text-cuts, and 377 pages of text. Philadelphia, New York, London: W. B. Saunders & Company. 1905. (Cloth, $3.50 net.)

Professor Haab's atlas and epitome of operative ophthalmology is the latest to appear in Saunders's reproductions of the medical hand-atlases of Lehmann, of Münich. In accuracy, pictorial beauty and compactness it is equal to any number of the series. It is a work more or less elementary in character, the first part dealing with the general consideration of hospital wards and operating rooms, anesthesia, antiseptics and asepsis, bandaging, disinfection and eye instruments; the second part, with all the important operations on the eye. In a concise manner it tells the student how to perform these operations, and accompanying this description are 154 text-cuts and 30 colored lithographic plates of most beautiful execution, which serve to make clear the author's ideas and his technic. To those who know the distinguished author personally, it is not necessary to speak of the painstaking and scientific character of the work; but to those who do not know him, it may be said that the subjects are treated with unexcelled clearness and accuracy. The teachings and technic are those of an experienced and skilful operator, and the faithfulness of description does not omit the minutest details of every important operation on the eye, including the after-treatment. Our own distinguished countryman, Dr. de Schweinitz, has had the editorial charge of the American edition, and this is a sufficient guarantee that the author's meaning has been carefully presented. He has also added some comments, and described a few operations not mentioned by Professor Haab, all of which contribute to the value of the book. While, as the author says, "The text has been condensed until it contains only what is indispensable," yet it is sufficiently extended to make it one of the best works on operations of the eye that can be placed in the hands of beginners in ophthalmological practice, especially of those who may not have the opportunity of frequently witnessing operations on the eye as performed by others.

This atlas is a fitting companion to the two previously published by Professor Haab—namely, that of "Ophthalmoscopy" and that of "External Diseases of the Eye," and the three taken together, constitute an enduring monument to the indefatigable and conscientious labors of this eminent teacher and practitioner. They should grace the library of every ophthalmologist.

